# Key regulators of lipid metabolism drive endocrine resistance in invasive lobular breast cancer

**DOI:** 10.1186/s13058-018-1041-8

**Published:** 2018-09-04

**Authors:** Tian Du, Matthew J. Sikora, Kevin M. Levine, Nilgun Tasdemir, Rebecca B. Riggins, Stacy G. Wendell, Bennett Van Houten, Steffi Oesterreich

**Affiliations:** 1 Women’s Cancer Research Center, UPMC Hillman Cancer Institute, Magee Womens Research Institute, 204 Craft Avenue, Pittsburgh, PA 15213 USA; 2 School of Medicine, Tsinghua University, Beijing, 100084 China; 3 Department of Pathology, University of Colorado Anschutz Medical Campus, Aurora, CO 80045 USA; 4 Department of Pathology, University of Pittsburgh, Pittsburgh, PA 15213 USA; 5 Department of Pharmacology and Chemical Biology, University of Pittsburgh, Pittsburgh, PA 15213 USA; 6 Department of Oncology, Georgetown-Lombardi Comprehensive Cancer Center, Georgetown University Medical Center, Washington, DC 20057 USA

**Keywords:** Invasive lobular breast, Endocrine resistance, LTED, SREBP1, Fatty acid, Cholesterol

## Abstract

**Background:**

Invasive lobular breast carcinoma (ILC) is a histological subtype of breast cancer that is characterized by loss of E-cadherin and high expression of estrogen receptor alpha (ERα). In many cases, ILC is effectively treated with adjuvant aromatase inhibitors (AIs); however, acquired AI resistance remains a significant problem.

**Methods:**

To identify underlying mechanisms of acquired anti-estrogen resistance in ILC, we recently developed six long-term estrogen-deprived (LTED) variant cell lines from the human ILC cell lines SUM44PE (SUM44; two lines) and MDA-MB-134VI (MM134; four lines). To better understand mechanisms of AI resistance in these models, we performed transcriptional profiling analysis by RNA-sequencing followed by candidate gene expression and functional studies.

**Results:**

MM134 LTED cells expressed ER at a decreased level and lost growth response to estradiol, while SUM44 LTED cells retained partial ER activity. Our transcriptional profiling analysis identified shared activation of lipid metabolism across all six independent models. However, the underlying basis of this signature was distinct between models. Oxysterols were able to promote the proliferation of SUM44 LTED cells but not MM134 LTED cells. In contrast, MM134 LTED cells displayed a high expression of the sterol regulatory element-binding protein 1 (SREBP1), a regulator of fatty acid and cholesterol synthesis, and were hypersensitive to genetic or pharmacological inhibition of SREBPs. Several SREBP1 downstream targets involved in fatty acid synthesis, including *FASN*, were induced, and MM134 LTED cells were more sensitive to etomoxir, an inhibitor of the rate-limiting enzyme in beta-oxidation, than their respective parental control cells. Finally, *in silico* expression analysis in clinical specimens from a neo-adjuvant endocrine trial showed a significant association between the increase of *SREBP1* expression and lack of clinical response, providing further support for a role of SREBP1 in the acquisition of endocrine resistance in breast cancer.

**Conclusions:**

Our characterization of a unique series of AI-resistant ILC models identifies the activation of key regulators of fatty acid and cholesterol metabolism, implicating lipid-metabolic processes driving estrogen-independent growth of ILC cells. Targeting these changes may prove a strategy for prevention and treatment of endocrine resistance for patients with ILC.

**Electronic supplementary material:**

The online version of this article (10.1186/s13058-018-1041-8) contains supplementary material, which is available to authorized users.

## Background

Accounting for 10–15% of all breast cancers, invasive lobular breast carcinoma (ILC) is the second most common histological subtype of breast cancer after invasive ductal cancer (IDC) [1, 2]. Tumor cells in classic ILC are small and round and invade the stroma in a discohesive single-file pattern, which can be attributed largely to the loss of E-cadherin (*CDH1*) [1]. Comparative analysis of luminal A ILC and IDC identified genomic and transcriptional differences between the two histological subtypes, including frequency of FOXA1 and GATA3 mutations, and activation of immune and metabolism pathways [3, 4]. ILC generally exhibits low rates of Ki67 and HER2 positivity, and more than 90% of ILC tumors are estrogen receptor–positive (ER^+^) [1–3]. Paradoxically, despite these favorable prognostic and predictive features, there is accumulating evidence that some patients with ILC have worse long-term survival compared with stage/grade-matched IDC [5–9].

Anti-estrogen therapy, targeting ER signaling, is an important part of the treatment for patients with ER^+^ breast cancer; however, its efficacy is often limited by intrinsic and acquired endocrine resistance. Mediators of endocrine resistance include loss of expression or genomic aberration (for example, mutations) of ER, altered expression of ER co-regulators and cell cycle signaling molecules, increased signaling of growth factor receptor pathways, and enhanced autophagy [10–12]. There have been only a limited number of studies testing the mechanism of anti-estrogen resistance in ILC, identifying potential roles for signaling through estrogen-related receptor gamma (ERRγ) [13], ERβ [14], FGFR1 [15, 16], and MAPK1 [17]. However, mechanisms of endocrine resistance in this understudied subtype of breast cancer remain largely unknown.

We have recently described a set of genes that are uniquely estrogen-regulated in ILC cells [15] and specifically identified WNT4 as an important mediator of estrogen-induced growth in ILC cells [18]. To test whether WNT4 played a similar role in anti-estrogen resistance in ILC, we generated long-term estrogen-deprived (LTED) MDA-MB-134VI (MM134) and SUM44PE (SUM44) ILC cell lines to mimic resistance to aromatase inhibitors (AIs) in the clinic and subsequently showed WNT4 overexpression in a subset of these models [18]. The objective of this study was to perform a comprehensive and unbiased characterization of these ILC LTED cell line models with the goal of identifying potential mechanisms of resistance. Here, we show that ILC LTED cells activate drivers of fatty acid/cholesterol metabolism, which could have therapeutic consequences and thus should pave the way for additional studies on unique cellular metabolism in lobular breast cancer.

## Methods

### Cell culture and reagents

SUM44PE (Asterand Bioscience, Detroit, MI, USA) cells were maintained as described previously [15]. MDA-MB-134VI (MM134) (American Type Culture Collection [ATCC], Manassas, VA, USA) cells were cultured in 1:1 Dulbecco’s modified Eagle’s medium (DMEM) (11,965; Life Technologies, Carlsbad, CA, USA): L-15 (11,415; Life Technologies) + 10% fetal bovine serum (FBS) (26,140; Life Technologies). LTED cell lines were generated as recently described [18]. Briefly, SUM44PE cells were cultured in 1:1 DMEM: L-15 + 10% FBS for 3 months to generate SUM44F cells, which have a stronger proliferative response to 17β-estradiol (E2) [18]. SUM44F and MM134 cells were hormone-deprived and then maintained in improved minimal essential medium (IMEM) (A10488; Life Technologies; Richter’s modification, no Phenol Red, no Gentamycin) + 10% charcoal-stripped FBS (CSS) (12,676, lot 1,747,185; Life Technologies) for 6–12 months to acquire endocrine resistance (Additional file 1: Figure S1). Four MM134 LTED variants (LTED-A, -B, -D, and -E) and two SUM44 LTED variants (LTED-A and -B) were generated independently. SUM44F was used as the parental cell line for SUM44 LTED cells in this study. CSS lot 1,747,185 was used for the majority of the experiments herein, but owing to unavailability of the same lot after its depletion, some studies were performed using FBS that was charcoal-stripped in our laboratory using a previously described methodology (26,140, lot 1,715,928; Life Technologies) [19]. The majority of experiments were repeated in both CSS lots to ensure consistent phenotypes. All cell lines were incubated at 37 °C in 5% carbon dioxide. Cell lines were authenticated at the University of Arizona Genetics Core and confirmed to be mycoplasma-negative with a MycoAlert Mycoplasma Detection Kit (LT07; Lonza, Basel, Switzerland). Authenticated cells were in continuous culture for less than 8 months (except during the establishment of LTED models).

For hormone deprivation, cells were washed twice daily for 3 days. For each wash, cells were rinsed twice with serum-free IMEM and then cultured in IMEM + 10% CSS. A minimum 1-h interval was kept between two washes.

E2 (E8875; Sigma-Aldrich, St. Louis, MO, USA), 25-hydroxycholesterol (25-HC) (SC-214091; Santa Cruz, Dallas, TX, USA), and 27-hydroxycholesterol (27-HC) (SC-358756; Santa Cruz) were dissolved in ethanol. PF429242 (SML0667; Sigma-Aldrich) was dissolved in double-distilled water (ddH_2_O). ICI 182,780 (ICI/fulvestrant) (1047; Tocris Bioscience, Avonmouth, Bristol, UK), etomoxir (E1905; Sigma-Aldrich), orlistat (O4139; Sigma-Aldrich), Fatostatin (F8932; Sigma-Aldrich), and TOFA (T6575; Sigma-Aldrich) were dissolved in dimethylsulfoxide (DMSO) (4-X; ATCC).

### Growth assays, dose response, and two-dimensional colony formation

For growth assays, 16,000 cells were seeded per well in 96-well plates. Parental cells were hormone-deprived before seeding. Day 0 was set as 24 h after seeding. Cells were harvested each day from day 0 to day 5. For dose response, cells were treated with vehicle (control) or a different concentration of drugs 24 h after seeding. Cell proliferation was quantified by using the Fluoreporter double-stranded DNA quantification kit (F2692; Life Technologies) in accordance with the instructions of the manufacturer. Fluorescence was assessed by using a VICTOR X4 plate reader (PerkinElmer, Waltham, MA, USA). The half maximal inhibitory concentration (IC_50_) was quantified with GraphPad Prism version 5.04 (GraphPad Software, La Jolla, CA, USA) using non-linear regression (log(inhibitor) versus response; three parameters).

For the two-dimensional (2D) colony formation, 5,000 cells per well were evenly seeded in six-well plates, media was changed once per week, and cells were harvested on day 15. Cells were washed twice with cold phosphate-buffered saline (PBS) and then fixed for 10 min with ice-cold 100% methanol. Fixed cells were stained with 0.5% crystal violet (C3886; Sigma-Aldrich. Dissolved in 25% methanol) for 10 min and further washed with H_2_O. Pictures of each well were taken with a SZX16 Stereo Microscope (Olympus, Tokyo, Japan). Colonies with radium of more than 25 μM were counted with cellSens Dimension version 1.9 (Olympus).

### RNA interference

Small interfering RNA (siRNA) was reverse-transfected into cells 24 h after cell seeding using Lipofectamine RNAiMAX transfection reagents (13,778,150; Life Technologies) in accordance with the instructions of the manufacturer. Specifically, for each well in a 96-well plate, cells were treated with 1 pmol *SREBP1* siRNA and 1 pmol *SREBP2* siRNA or with 2 pmol non-target siRNA. SiRNA sequences are provided in Additional file 2: Table S1.

### Q-RT-PCR

RNA was extracted with a Qiagen RNeasy kit (74,106; Qiagen, Hilden, Germany). iScript reverse transcription supermix (1,708,841; Bio-Rad Laboratories, Hercules, CA, USA) was used to generate cDNA. Quantitative polymerase chain reaction (PCR) was then carried out with a CFX384 Real-Time PCR Detection System (Bio-Rad Laboratories) using SsoAdvanced SYBR Green Master Mix (Bio-Rad Laboratories). *RPLP0* was used as the internal control to normalize gene expression. Primer sequences are provided in Additional file 2: Table S1.

### Immunoblotting

For whole cell lysis, cells were lysed with RIPA buffer supplied with Halt Protease and Phosphatase inhibitor (78,842; Thermo Fisher Scientific, Waltham, MA, USA). Nuclear proteins were extracted with NE-PER™ Nuclear and Cytoplasmic Extraction Reagents (78,833; Thermo Fisher Scientific) in accordance with the instructions of the manufacturer. Proteins were separated by SDS-PAGE and transferred to polyvinylidene difluoride (PVDF) membranes. Protein bands were detected by fluorescence with Odyssey CLX imaging system (LI-COR Biosciences, Lincoln, NE, USA). The following primary antibodies were used: anti-ERα (8644; Cell Signaling Technology, Danvers, MA, USA; dilution 1:1000), anti-SREBP1 (SC-13551; Santa Cruz; dilution 1:200), anti-β-actin (A5441; Sigma-Aldrich; dilution 1:2500), and anti-FASN (3180S; Cell Signaling Technology; dilution 1:1000). Anti-PCNA (NA03; EMD Millipore, Billerica, MA, USA; dilution 1:1000) was kindly provided by Yi Huang (UPMC Hillman Cancer Center) and used as the internal control for nuclear protein.

### RNA-sequencing and differential expression analysis

Parental and LTED MM134 and SUM44 cells were seeded in triplicates in six-well plates. Parental cells were hormone-deprived for 3 days before cell collection. RNA was isolated by using an Illustra RNAspin Mini Kit (25–0500-72; GE Healthcare, Little Chalfont, UK). RNA-sequencing (RNA-Seq) was carried out by Illumina HiSeq 2000. Raw sequence data were mapped to hg38 genome (ensemble release version 82) and gene counts were quantified with Salmon (version 0.6.0) [20] using default settings. RNA-Seq mapping rates are provided in Additional file 3: Table S2. Differentially expressed (DE) analysis was performed with R package DESeq2 [21] in MM134 cells and SUM44 cells independently. DE genes in individual LTED variants were called using the following criteria: absolute log2(fold change) > log2(1.5) and Benjamini-Hochberg–adjusted *P* value of less than 0.001. The complete list of DE genes is available in Additional file 4: Table S3. RNA-Seq raw sequence data are available via GSE116744 from gene expression omnibus (GEO) (http://ncbi.nlm.nih.gov/geo/).

The gene expression (microarray) data of SUM44 tamoxifen-resistant (SUM44 TamR) and parental cells (SUM44PE) were downloaded from GEO [GSE12708]. Probes with the highest interquartile range were selected for genes that matched to multiple probes. DE analysis was performed with R package Limma [22], and a Benjamini-Hochberg–adjusted *P* value of less than 0.05 was used to call DE genes in SUM44 TamR cells.

### Heatmap clustering

The Salmon output of gene-level transcript per million (TPM) counts was used, first transforming by log2 (TPM + 1). The top 1000 most variable genes in MM134 or SUM44 cells (by interquartile range) were used for the heatmap. Relative expression values were calculated as fold change to the average expression level in parental cells. Hierarchical clustering of genes was conducted by using the heatmap.3 function (https://raw.githubusercontent.com/obigriffith/biostar-tutorials/master/Heatmaps/heatmap.3.R) under R version 3.2.2. The relationship between genes in terms of expression patterns across different samples was quantified with a Euclidean distance measure and visualized with complete-linkage clustering.

### Pathway analysis

Pathway analysis was conducted with Ingenuity Pathway Analysis (IPA) using genes that were differentially expressed in at least three MM134 LTED variants or both SUM44 LTED variants. Complete pathway analysis results are shown in Additional file 5: Table S4. *GseaPreranked* function in Gene Set Enrichment Analysis (GSEA) (version 2.2.2, Broad Institute, Cambridge, MA, USA) was performed using the Reactome cholesterol synthesis signature (Additional file 5: Table S4), downloaded from the Molecular Signature Database (MsigDB, version 6.0, Broad Institute). DE genes ranked by their log2(fold change) were used as input. Default settings in *GseaPreranked* were used except the following parameters: “Enrichment statistic” was “Classic” and “Min size: exclude smaller sets” was set to be 0.

### LC-MS/MS analysis

Cells were washed twice with cold PBS, scratched off the plate, and resuspended in cold PBS, and cell suspensions for each sample were spiked with cholesterol-d_7_ (1.27 nmoles) and 16:0-cholesterol ester-d_7_ (1.58 nmoles) internal standards and extracted using two volumes of isopropanol/chloroform/formic acid (50/50/0.1). Samples were centrifuged at 3000 revolutions per minute at room temperature for 10 min. The bottom layer (organic) was transferred to a clean vial and dried under N_2_. Samples were reconstituted in 150 μL of chloroform/methanol (1:2) for high-performance liquid chromatography-electrospray ionization tandem mass spectrometry analysis (LC-MS/MS).

Samples were analyzed on a Sciex 5000 triple quadrupole coupled to a Shimadzu/CTC Leap HPLC system (Sciex, Framingham, MA, USA). Cholesterol and metabolites (for example, 27-HC) were separated by a Luna C18(2) reversed-phase column (5 μ, 2 X 100 mm; Phenomenex, Torrence, CA, USA) at a flow rate of 0.63 mL/min using a linear gradient. Solvent A consisted of water/acetonitrile/formic acid (50/50/0.1), and solvent B consisted of acetonitrile/iso-propanol/formic acid (40/60/0.1). The gradient started at 50% B and increased over 6 min to 100% B, which was maintained for 5 min and followed by a 4-min equilibration at initial conditions. Cholesterol esters were separated using the same column and flow rate, but the solvent system consisted of water/acetonitrile/formic acid (10/90/0.1) for solvent A and acetonitrile/isopropanol//formic acid (20/80/0.1) for solvent B. The gradient started at 55% B and increased over 9 min to 100% B, which was maintained for 4 min and followed by a 4-min equilibration at initial conditions. Samples were analyzed in positive ion mode using the following MS source and compound parameters: collision gas 5, curtain gas 40, ion source gas 1 55, ion source gas 2, 50, ion spray voltage 5500, temperature 600 °C, de-clustering potential 90, entrance potential 5, collision exit potential 10, and a collision energy of 25. The following transitions were used for cholesterol, 27-hydroxylcholesterol, and cholesterol esters: 369.3➔161.3 cholesterol and cholesterol esters, 376.4➔161.3 cholesterol-d_7_ and 16:0-cholesterol ester-d_7_, and 385.3➔215.1 27-hydroxylcholesterol. Cholesterol was quantified by using a standard curve developed with standards and the cholesterol-d_7_ internal standard. Cholesterol esters were reported by integrating the total peak areas for cholesterol ester species detected and quantified using the 16:0-cholesterol ester and 16:0-cholesterol ester-d_7_ standard curve. Cholesterol, 27-hydroxylcholesterol, and cholesterol esters are reported as nanomoles per 1000,000 cells for cell lysate.

### Analysis of clinical samples

Gene expression microarray data of ER^+^ breast cancers treated with letrozole were obtained from the GEO database [GEO: GSE20181] [23]. Gene expression changes after 3-month treatment with letrozole were determined for genes of interest in responders and non-responders, as defined by tumor volume reduction by 50%. Pearson’s chi-squared test was used to check the dependence of the gene expression change and the response to letrozole.

## Results

### Growth and hormone response of MM134 and SUM44 LTED cells

**Fig. 1:**
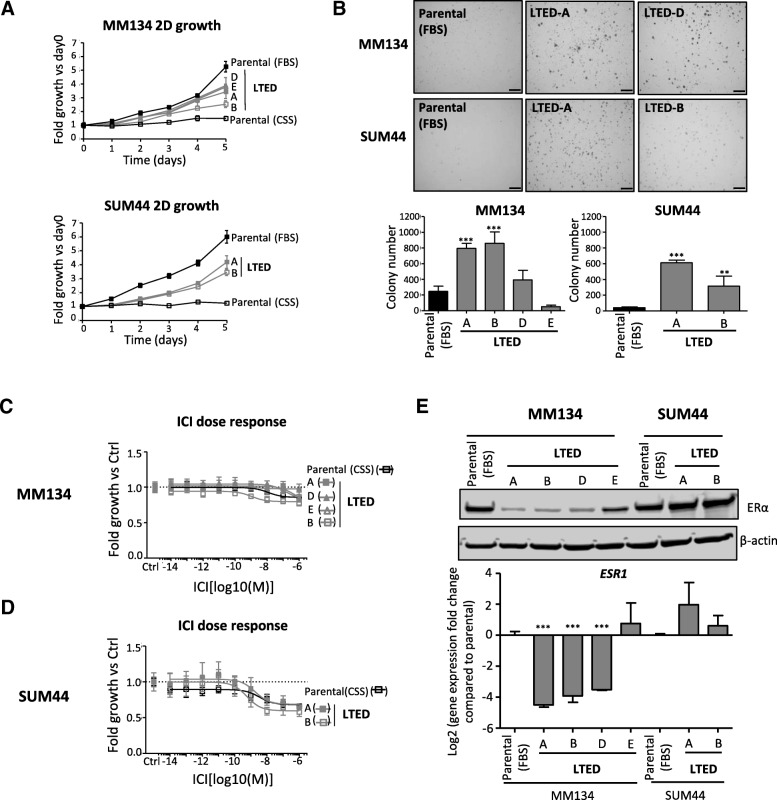
MM134 and SUM44 long-term estrogen deprivation (LTED) cells have different estrogen receptor (ER) activity. **a** Growth curve of LTED and their parental cells. Parental cells were cultured in either their normal growth media (FBS, 1:1 DMEM:L-15 + 10% FBS) or the hormone-deprived media (CSS, IMEM + 10% CSS). Parental cells in the CSS group were hormone-deprived before seeding. “(FBS)” and “(CSS)” were used to represent the normal growth media and hormone-deprived media, respectively, throughout the article. SUM44F served as the parental cell line for SUM44 LTED cells in the article. Plots are representative of three independent experiments. Data are mean ± standard deviation (SD) of six replicates. **b** Two-dimensional (2D) colony formation of parental (grown in FBS) and LTED (grown in CSS) cells. Pictures of MM134 LTED-A and LTED-D colonies were selected as representative pictures for MM134 LTED cell lines. Five thousand cells were seeded per well in six-well plates, and cells were stained with crystal violet after 15 days. The colonies with radium of more than 25 μm were counted for the colony numbers. Scale bar, 1 mm. Plots are representative of two independent experiments. Data are mean ± SD of three replicates. One-way analysis of variance (ANOVA) followed by Dunnett’s post hoc test for multiple comparisons, **P <0.01, ***P <0.001. (**c**, **d**) Dose response ICI 182, 780 (ICI) in MM134 (**c**) and SUM44 (**d**) LTED cells. Cells were treated with vehicle (Ctrl) or drugs for 5 days before collection. Plots are representative of at least two independent experiments. Data are mean ± SD of six replicates. **e** ERα (Western blot; top) and *ESR1* (quantitative reverse transcription-polymerase chain reaction, or qRT-PCR; bottom) expression in MM134 and SUM44 LTED and parental cells. Data in the lower panel are mean ± SD of three replicates. One-way ANOVA followed by Dunnett’s post hoc test for multiple comparisons, ***P <0.001. Abbreviations: *CSS* charcoal-stripped fetal bovine serum, *DMEM* Dulbecco’s modified Eagle’s medium, *FBS* fetal bovine serum, *IMEM* improved minimal essential medium.

The MM134 and SUM44 LTED cells were generated by growing parental cells in hormone-deprived serum (IMEM + 10% charcoal-stripped FBS, CSS) over 6–12 months until the emergence of several estrogen-independent clones, which were subsequently maintained in this medium [18] (Additional file 1: Figure S1). Growth analyses showed that all of the LTED clones had significantly increased growth rates as compared with their parental lines grown in CSS (Parental [CSS]) (Fig. 1a), confirming their estrogen-independent growth. While the LTED cells proliferated more slowly than their respective parental lines grown in FBS (Parental [FBS]) over 5 days (Fig. 1a), 3/4 MM134 and 2/2 SUM44 LTED clones showed significantly increased growth or colony number (or both) in colony formation assays over 15 days, suggesting that LTED cells have higher clonogenic ability (Fig. 1b). Neither parental cells nor LTED variants, however, were able to form colonies in soft agar when grown in CSS (Additional file 1: Figure S2A). These results are likely due to different signaling pathways governing 2D and 3D growth in CSS versus FBS.

To comprehensively characterize the endocrine response of the LTED cell lines, we performed dose response assays with E2 and ICI 182,780 (ICI). These growth assays showed that MM134 and SUM44 LTED cells do not respond to E2 (Additional file 1: Figure S2B). In SUM44 LTED cells, we observed some weak growth inhibition with estradiol; however, this was a weak effect that varied with different lots of CSS (Additional file 1: Figure S2B, bottom panel). ICI had no effect on MM134 LTED (Fig. 1c) but did result in growth inhibition of SUM44 LTED cells (Fig. 1d). These results were supported by analysis of ER protein levels in MM134 and SUM44 LTED cells, showing decreased and increased ER expression compared with their parental cells, respectively (Fig. 1e). Collectively, these data suggest that the two ILC LTED cell line models differ in their hormone response; MM134 LTED cells had very low ER protein levels and lacked a hormone response, whereas SUM44 LTED cells maintained high ER expression and had some response to ICI.

### Activation of lipid synthesis-related pathways in ILC LTED cells

**Fig. 2:**
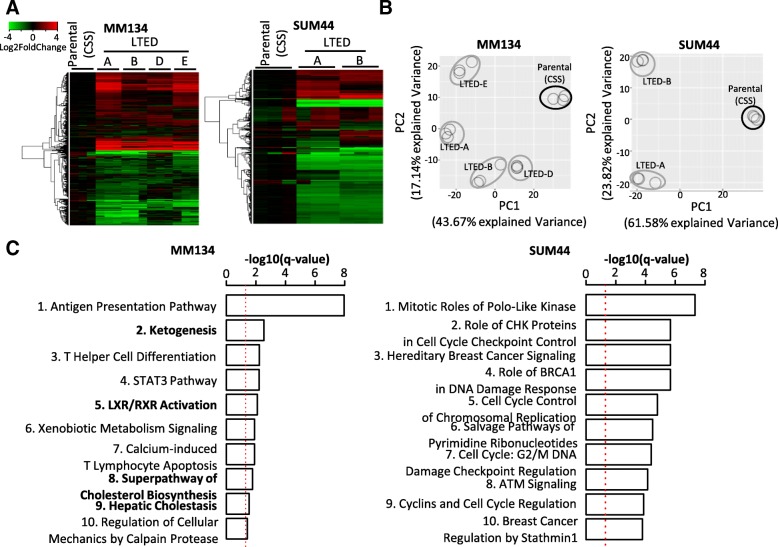
MM134 and SUM44 long-term estrogen deprivation (LTED) cells show different top enriched pathways. (**a**, **b**) Heatmaps (**a**) and principal component analysis (PCA) plots (**b**) of the top 1000 most variable genes. The top 1000 most variable genes were selected using interquartile range (IQR) in MM134 or SUM44 cells independently. The clustering of genes in heatmaps was based on complete-linkage, Euclidean distance hierarchical clustering. **c** Top 10 upregulated pathways in MM134 or SUM44 LTED cells. Ranked by –log10(*P* value). –log10(0.05) is marked with a red line. Cholesterol- and fatty acid-related pathways are labeled in bold. Pathway analyses were performed with Ingenuity Pathway Analysis (IPA) with the commonly upregulated differential expression (DE) genes in at least three MM134 LTED variants (*n* = 1653) or in the two SUM44 LTED variants (*n* = 1448). *P* values were corrected with the Benjamini-Hochberg method

To better understand the gene expression and pathway changes in LTED cells, we performed transcriptional profiling by RNA-Seq on LTED clones and their respective parental cells. Heatmap (Fig. 2a) and principal component analysis (Fig. 2b) showed that the different LTED variants were similar to each other and distinct from their parental cells by gene expression. Three thousand three hundred fifty-nine DE genes (upregulated *n* = 1653 and downregulated *n* = 1706) were shared in at least three MM134 LTED cell lines (Additional file 1: Figure S3A; Additional file 4: Table S3), and 2106 genes were shared in all four lines (*P* <2.2e-16). Three thousand two hundred sixteen DE genes (upregulated *n* = 1448, downregulated *n* = 1768) were shared by the two SUM44 LTED variants (*P* <2.2e-16) (Additional file 1: Figure S3B; Additional file 4: Table S3). Whereas MM134 LTED and SUM44 LTED cells shared a significant number of DE genes (*n* = 437, *P* <2.2e-16), the majority of DE genes were unique for MM134 LTED or SUM44 LTED cells (Additional file 1: Figure S3C), indicating that the two cell line models have acquired some shared but also many different mechanisms of endocrine resistance.

**Fig. 3:**
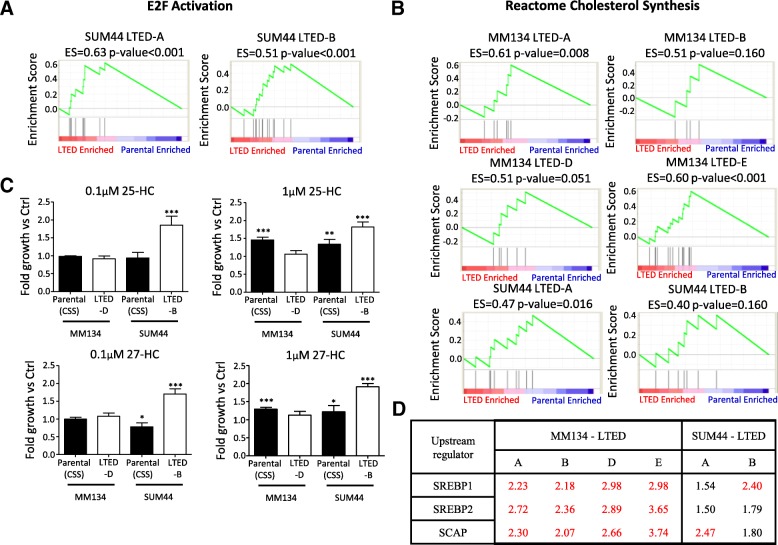
Cholesterol synthesis is predicted to be upregulated in long-term estrogen deprivation (LTED) cells. **(a, b)** Gene set enrichment analysis (GSEA) of (**a**) E2F activation signature and (**b**) cholesterol biosynthesis signature (Reactome Cholesterol Synthesis) in LTED variants. Differential expression (DE) genes used in GSEA were ranked by log2(fold change). **c** Growth of parental and LTED cells with treatment of 25-hydroxycholesterol (25-HC) and 27-hydroxycholesterol (27-HC). Parental cells were hormone-deprived before being seeded in hormone-deprived media (charcoal-stripped fetal bovine serum, or CSS). Cells were collected after 5-day treatment. Fold growth was compared with control group (data not shown), which was treated with vehicle (ethanol). Plots are representative of at least two independent experiments. Data are mean ± standard deviation (SD) of six replicates. One-way analysis of variance (ANOVA) followed by Dunnett’s post hoc test for multiple comparisons was used to test the significance between 25-HC/27-HC–treated groups to the control groups (data not shown), *P <0.05, **P <0.01, ***P <0.001. **d** The activation z-score of sterol regulatory element-binding proteins (SREBPs) in LTED cells. The upstream regulator analysis was performed in individual LTED cell variants separately with Ingenuity Pathway Analysis (IPA) software. DE genes (absolute log2(fold change) > log2(1.5) and adjusted *P* value of less than 0.001) and their log2(fold change) were used as input. Upstream regulators with z-score of more than 2 were defined as “activated” (labeled in red). Abbreviation: *ES* enrichment score.

To identify pathways activated in the two resistant models, we applied IPA using the DE genes from MM134 and SUM44 LTED cells (Fig. 2c, Additional file 5: Table S4). The most strongly enriched pathways in MM134 versus SUM44 were distinct, again suggesting at least some differences in mechanisms of endocrine resistance. In MM134 LTED cells, the most enriched pathways were related to activation of immune functions and metabolism. Intriguingly, the enriched metabolic pathways were all related to lipid synthesis and lipid metabolism, such as “Ketogenesis” and “Superpathway of Cholesterol Biosynthesis” (indicated in bold in Fig. 2c). Similar pathways were not the primary pathways enriched in SUM44 LTED cells, which in contrast showed activation of DNA repair mechanisms and pathways related to cell cycle checkpoints. This finding was supported by the results from the GSEA, showing significant enrichment of an E2F signature in SUM44 LTED cells (Fig. 3a) but not in MM134 LTED cells (data not shown). These data suggest that one mechanism of endocrine resistance in SUM44 LTED cells is E2F-mediated activation of cell proliferation, previously described for other endocrine-resistant models [24].

We noted that although cholesterol-related signatures were not among the top 10 activated pathways in SUM44 LTED cells, the “Superpathway of Cholesterol Biosynthesis” pathway was still significantly enriched in this model (*P* = 0.023, corrected *P* = 0.18). A possible activation of cholesterol synthesis in MM134 and SUM44 LTED cells was further suggested by results from GSEA, which identified a modest enrichment of a cholesterol synthesis signature (Fig. 3b).

“Ketogenesis” involves β-oxidation of fatty acids [25], whereas the “Superpathway of cholesterol biosynthesis”, “LXR/RXR Activation”, and “Hepatic Cholestasis” are closely associated with both cholesterol and fatty acid transport and metabolism [26, 27]. We did not detect differences in total intracellular free cholesterol and cholesterol esters between parental MM134 or SUM44 and LTED cells (Additional file 1: Figure S4). As cholesterol metabolites (for example, 27-HC) were increased in endocrine-resistant IDC cells [28, 29], we investigated their potential role in our ILC LTED models. Although we were unable to quantify 27-HC because the levels were below the limit of detection in our assays, growth assays showed that the cholesterol metabolites 25-HC and 27-HC could promote the proliferation of SUM44 LTED cells, as well as MM134 and SUM44 parental cells grown in CSS, suggesting a potential similar role of cholesterol metabolites in endocrine-resistant ER^+^ ILC models (Fig. 3c).

### Upregulation of sterol regulatory element-binding factor SREBP1 and other fatty acid synthesis enzymes in LTED ILC cells

**Fig. 4:**
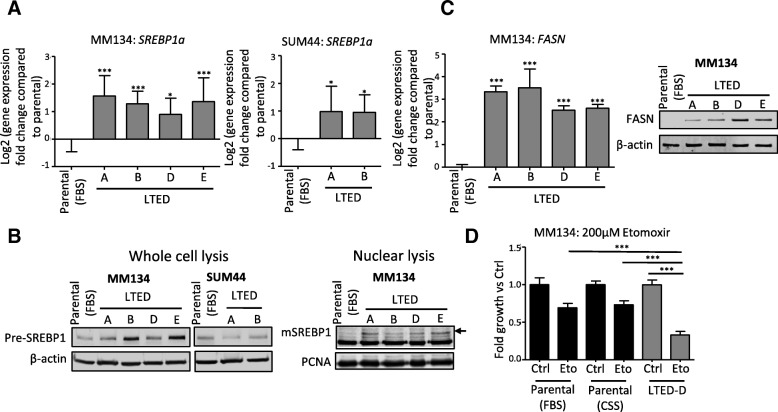
Cholesterol synthesis regulator, sterol regulatory element-binding protein 1 (SREBP1) is upregulated in long-term estrogen deprivation (LTED) cells. **a** Quantitative reverse transcription-polymerase chain reaction (qRT-PCR) of *SREBP1a* in LTED and parental cells. Parental cells were cultured in their normal growth media (fetal bovine serum, or FBS). Plots are the combination of data from three independent experiments. Data are mean ± standard deviation (SD) of nine replicates. One-way analysis of variance (ANOVA) followed by Dunnett’s post hoc test for multiple comparisons, *P <0.05, **P <0.01, ***P <0.001. **b** The expression of precursor SREBP1 (pre-SREBP1) and mature SREBP1 (mSREBP1) in parental and LTED cells. Because of their similar molecular weight (54 amino acid difference in length), SREBP1a and SREBP1c were not separated in the gel. β-actin and PCNA were used as internal control in the whole cell lysis and nuclear lysis, respectively. The band of mSRBP1 is labeled with an arrow. Blots of whole cell lysis are representative of three independent experiments, and the blot of nuclear lysis is a single experiment. **c** RNA and protein expression levels of fatty acid synthase (*FASN*). Plots are representative of two independent experiments. Data in the left panel are mean ± SD of three replicates. One-way ANOVA followed by Dunnett’s post hoc test for multiple comparisons, *P <0.05, **P <0.01, ***P <0.001. **d** The growth inhibition of MM134 parental and LTED cells by etomoxir. MM134 LTED-D was selected as the representative variant of MM134 LTED cells. This figure is the same experiment as the dose response curve of etomoxir in Additional file 1: Figure S8B. Plot is representative of two independent experiments. Data are mean ± SD of six replicates. One-way ANOVA followed by Dunnett’s post hoc test for multiple comparisons, ***P <0.001

Lipid homeostasis is closely regulated by sterol regulatory element-binding factors (SREBPs/SREBFs) [30, 31]. The SREBP family that has been proposed to have roles in tumor differentiation, metastasis, and dormancy [32–34] contains two genes, *SREBP1* and *SREBP2*, which encode three protein isoforms: SREBP1a, SREBP1c, and SREBP2. Functionally, SREBP1c and SREBP2 regulate fatty acid synthesis and cholesterol synthesis, respectively, while SREBP1a regulates both of these pathways [35, 36]. We identified *SREBP1* as one of the only five genes (*SREBP1*, *FASN*, *FGFR4*, *AKR7A3*, and *FKBP11*) that were commonly upregulated in MM134 LTED, SUM44 LTED, and SUM44 TamR cells (previously described by Riggins et al. [13]). Further supporting a role for the SREBP family in endocrine resistance in ILC, IPA upstream regulator analysis showed that SREBP1, SREBP2, and SCAP (SREBP cleavage-activating protein) were activated in the ILC LTED models (Fig. 3d). Expression analysis of the three SREBP isoforms (SREBP1a, SREBP1c, and SREBP2) showed upregulation of *SREBP1a* in all MM134 and SUM44 LTED lines (Fig. 4a, and Additional file 1: Figure S5). At the protein level, the precursor SREBP1 (pre-SREBP1) was increased in MM134 LTED but not in SUM44 LTED cells (Fig. 4b, left panel). Pre-SREBP1 is processed into mature SREBP1 (mSREBP1), which activates the transcription of target genes by binding to the sterol response element in nucleus (Additional file 1: Figure S6). Our cell fractionation and immunoblot analyses showed upregulation of mSREBP1 in the nucleus in MM134 LTED cells compared with parental cells (Fig. 4b, right panel), supporting increased transcriptional activity in endocrine-resistant MM134 cells.

Given the known role of SREBP1 in fatty acid synthesis [30, 31], we asked whether FASN, a key enzyme regulating fatty acid synthesis and direct target gene of SREBP1, was also induced in LTED cells. RNA and protein analysis showed significant upregulation of *FASN* in all four MM134 LTED lines (Fig. 4c), and there was a trend toward higher expression in the SUM44 LTED cells (Additional file 1: Figure S7A). Significant *FASN* upregulation was also previously reported in SUM44 TamR cells [13]. Expression of other key enzymes in the fatty acid synthesis pathway (*ACACA*, *ACLY*, and *SCD*) was also upregulated but with variation among the different LTED cell lines (Additional file 1: Figure S7B).

FASN plays an important role in the *de novo* synthesis of long-chain fatty acids, which promotes the progression of tumors by fueling both membrane synthesis and energy production through β-oxidation [37, 38]. Since the pathway analysis also suggested activation of “Ketogenesis”, which involves the β-oxidation of fatty acids [25], we set out to determine whether the LTED cells were more dependent on fatty acid synthesis compared with their parental cells. Therefore, we treated the cells with various inhibitors of fatty acid synthesis and fatty acid oxidation. There was no significant difference between parental and LTED cells upon treatment with orlistat and TOFA, inhibitors of FASN and ACACA, respectively (Additional file 1: Figure S8A, B). However, MM134 LTED cells were significantly more sensitive to etomoxir, an inhibitor of carnitine palmitoyltransferase 1 (CPT-1), the rate-limiting enzyme in β-oxidation, than their parental cells (Fig. 4d, Additional file 1: Figure S8B). Collectively, these data suggest that the ILC LTED cell lines activate and induce a number of enzymes critical in fatty acid and cholesterol metabolism for energy production.

### Role for SREBPs in endocrine-resistant cell lines and in clinical samples

**Fig. 5:**
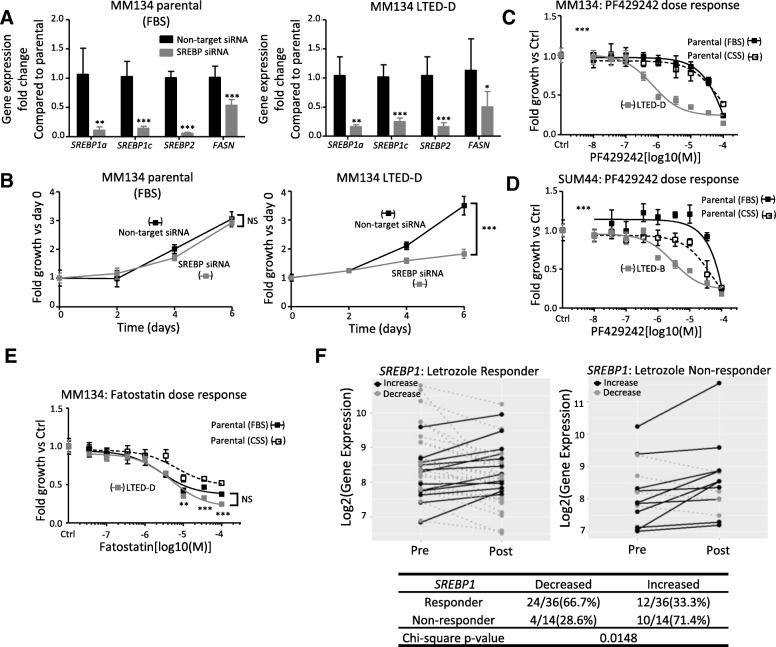
Abrogation of sterol regulatory element-binding proteins (SREBPs) inhibits the growth of long-term estrogen deprivation (LTED) cells. **a** Knockdown efficiency of *SREBP1* and *SREBP2* in MM134 parental and LTED-D cells. mRNA was collected 72 h after reverse transfection with small interfering RNA (siRNA). Data are mean ± standard deviation (SD) of six replicates collected from two independent experiments. Two-tailed Welch’s unequal variances *t* test, *P ≤0.05, **P <0.01, ***P <0.001. **b** Growth curve of MM134 parental and LTED cells with SREBP knockdown. Parental and LTED cells in 96-well plates were reverse-transfected with 1 nM siSREBP1 and 1 nM siSREBP2 (SREBP siRNA) or 2 nM non-target siRNA. Parental cells were cultured in their normal growth media (fetal bovine serum, or FBS). Two-way analysis of variance (ANOVA), ***P <0.001. **(c, d)** Dose response of PF429242, an inhibitor of SREBP1 and SREBP2, in MM134 (**c**) and SUM44 (**d**) parental and LTED cells. Parental cells were grown in normal growth media (FBS) and hormone-deprived media (charcoal-stripped fetal bovine serum, or CSS) to control the effect of media on the drug. Plots are representative of two independent experiments. Data are mean ± SD of six replicates. Two-way ANOVA (LTED versus parental in FBS), ***P <0.001. **e** Dose response of Fatostatin in MM134 parental and LTED cells. Parental cells were grown in normal growth media (FBS) or hormone-deprived media (CSS) to control the effect of media on the drug. Plots are representative of two independent experiments. Data are mean ± SD of six replicates. Two-way ANOVA was used to compare the dose response curves (LTED versus parental in FBS). Two-tailed *t* tests were performed to compare the inhibition rates of Fatostatin on LTED and parental (FBS) at 10 μm, 35 μm, and 100 μm independently. **P <0.01, ***P <0.001. **f** The expression change of *SREBP1* with 3-month letrozole treatment in letrozole responders (*n* = 36) and non-responders (n = 14). Black solid lines, increased expression of *SREBP1* with 3-month treatment; gray dashed lines, decreased expression of *SREBP1* with 3-month treatment. Gene expression data for letrozole-treated patients were downloaded from Gene Expression Omnibus [GSE20181]. Pearson’s chi-squared test. Abbreviation: *NS* not significant.

Next, we set out to directly assess the role of SREBP family members in the growth of the LTED cells. We selected two siRNA pools that target both genes (*SREBP1* and *SREBP2*) simultaneously, thereby inhibiting potential compensatory mechanisms. Successful knockdown was confirmed by quantitative reverse transcription-polymerase chain reaction (q-RT-PCR), which also showed that SREBP knockdown resulted in decreased levels of the target gene *FASN*, as expected (Fig. 5a). Decreased SREBP levels significantly inhibited the proliferation of MM134 LTED cells by more than 50% without having an effect in MM134 parental cells (Fig. 5b, Additional file 1: Figure S9C). MM134 LTED cells were also significantly more sensitive than their parental cells to PF429242 (Fig. 5c), an inhibitor of SREBP maturation. Similar siRNA knockdown studies were performed in SUM44 cells, which showed decreased growth in both parental and LTED cells (Additional file 1: Figure S9A–C). When treated with PF429242, the SUM44 LTED cells were more sensitive than their parental cells (Fig. 5d), although the effect is less pronounced compared with MM134 cells. Fatostatin, a drug preventing SCAP-mediated escort of SREBP and thus interfering with downstream transcriptional effects, also inhibited growth of MM134 LTED cells (Fig. 5e), with minimal effects in SUM44 LTED cells (Additional file 1: Figure S9D).

Finally, to assess potential clinical relevance of SREBPs in endocrine resistance, we measured *SREBP1* and *SREBP2* expression in breast tumors following estrogen deprivation therapy. Specifically, we performed *in silico* gene expression analysis in 50 primary ER^+^ breast cancers before treatment (“pre”) and after 3 months of treatment (“post”) with the AI letrozole [23]. Tumors with reduction greater than 50% or less than 50% in volume were classified as letrozole responders (*n* = 36) and non-responders (*n* = 14), respectively. Following the 3-month regimen, non-responders had a higher incidence of increased expression of *SREBP1* (Fig. 5f) compared with letrozole responders (10/14, 71.4% versus 12/36, 33.3%; Pearson’s chi-squared test, *P* value = 0.0148). *SREBP2* upregulation was not significantly different between letrozole responders and non-responders (Additional file 1: Figure S10). These data suggest that SREBP1 induction is associated with the development of resistance to estrogen deprivation therapy.

## Discussion

Resistance to endocrine therapy is a major limitation in the treatment of ER^+^ breast cancers. Although there is the increasing realization that ILC is a disease distinct from IDC in many features [1, 2, 4, 5, 39, 40], only a few studies have investigated endocrine resistance with ILC models [13, 18, 24, 41]. In this study, we comprehensively characterized a total of six ILC LTED variants from MM134 and SUM44PE (SUM44F), the two most commonly used ER^+^ ILC cell lines. To the best of our knowledge, we are the first group to generate endocrine-resistant cell models with MM134, a cell line that shows *de novo* tamoxifen resistance [15]. Of note, MM134 LTED cells are also resistant to ICI 182,780 (fulvestrant), which makes them a unique model to investigate ER-independent mechanisms of acquired endocrine resistance.

We found that MM134 and SUM44 LTED cells acquired some shared but also unique adaptive mechanisms of resistance to estrogen deprivation. SUM44 LTED cells showed activated E2F signaling, a pathway that was previously reported to be upregulated in endocrine-resistant IDC [24, 42]. This pathway was not activated in MM134 LTED cells that showed minimal ER expression, and had lost hormone response. Given the recent success of targeting the CDK-RB-E2F signaling pathway [43], SUM44 LTED cells might represent an excellent model for the study of CDK4/6 inhibitors in ER^+^ endocrine-resistant ILC, which we will test in future studies.

An adaptive mechanism of resistance that was shared between the two ILC LTED models was the activation of fatty acid and cholesterol metabolism pathways. High cholesterol level was reported to be a risk factor of the early recurrence in breast cancers [44]. The BIG 1-98 study [45] showed that cholesterol-lowering medication was related to improved clinical outcomes in early-stage hormone receptor–positive breast cancers, suggesting a potential role of cholesterol in causing endocrine resistance. Recent studies by Simigdala et al. [28] and Nguyen et al. [29] showed upregulation of cholesterol biosynthesis in AI-resistant breast cancer. In addition, Martin et al. [41] recently reported that SUM44 LTED cells have higher fatty acid dependency than their parental cells, which was hypothesized to be due to increased expression of fatty acid metabolism genes as a result of an *ESR1* mutation in this set of LTED cells. Of note, *ESR1* is not mutated in any of our ILC cell line models [41]. Although (at least in part owing to technical limitations) we were unable to detect higher levels of cholesterol, cholesterol esters, and oxysterols, we did observe an increased sensitivity of SUM44 LTED cells to 25-HC, which was not seen in MM134 LTED. These results are in line with the previously reported findings that oxysterols can directly bind to and activate ER [28, 46] and that, in the absence of estrogen, oxysterols can activate growth in an ER-dependent manner [28, 29]. However, oxysterols can also bind to other nuclear receptors such as farnesoid X receptor (FXR), liver X receptor (LXR), retinoic acid receptor-related orphan receptor (ROR), peroxisome proliferator-activated receptors (PPAR), and pregnane X receptor (PXR) [47, 48], and thus further studies are required to fully define the expression and action of oxysterols in the LTED cells.

A series of enzymes critically involved in fatty acid synthesis were induced in our ILC LTED models, especially FASN, which is known to be highly expressed in many human epithelial cancers and their pre-neoplastic lesions [38]. The upregulation of FASN has been linked to the acquisition of resistance to chemotherapy in breast and ovarian cancers [49–52]; however, there is limited information about an association between FASN and endocrine resistance. And whereas one study showed that FASN inhibition could reverse anti-estrogen resistance in MCF7 cells [53], others showed that FASN blockade increased the sensitivity of ER to E2 [54, 55] and thus there might be context-dependent effects that need to be elucidated further. Interestingly, two studies [56, 57] recently reported that endocrine therapy increases the risk of newly developed fatty liver, but it is not clear whether and how this might be linked to FASN expression in this setting. Of note, we did not observe increased sensitivity to fatty acid synthesis inhibitors, which might be due to the increased ability to use exogenous fatty acids when the *de novo* synthesis pathway is inhibited [58–60]. This has been reported in prostate cancer cells, which could be inhibited only by C75 and SB204990, inhibitors of FASN and ACLY, respectively, in the absence of lipoprotein, the transporter of exogenous fatty acid and cholesterol [61].

**Fig. 6:**
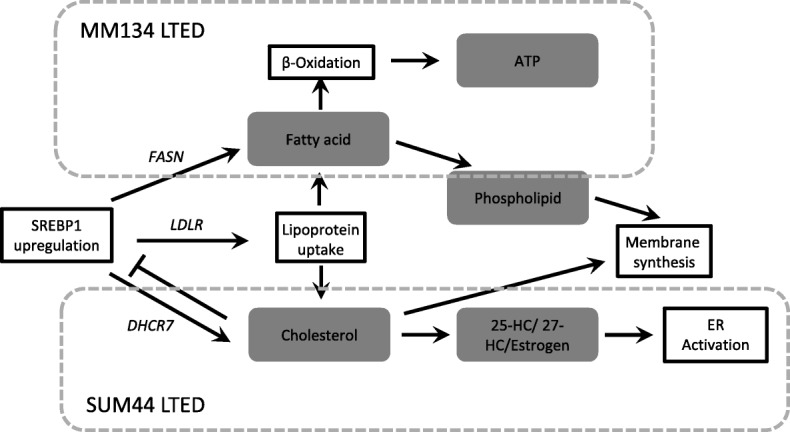
Proposed working model for role of sterol regulatory element-binding protein 1 (SREBP1) signaling in invasive lobular carcinoma (ILC) long-term estrogen deprivation (LTED) cells. Genes: the upregulated genes in MM134 or SUM44 LTED cells compared with parental cells, which were validated in the *in vitro* experiments or based on the RNA-sequencing (RNA-Seq) data or both. Black arrow, the potential pathway of SREBP1 promoting the survival of LTED cells

The ILC LTED cells showed increased sensitivity to genetic and pharmacologic inhibition of SREBP, and consistently stronger effects were seen in MM134 LTED cells. Similarly, gene expression changes were more pronounced in MM134 LTED cells. These data suggest that activation of lipid-metabolic pathways might not be related to activation of ligand-independent ER signaling but could drive fatty acid oxidation (FAO), membrane synthesis, or other processes (Fig. 6). In support of increased FAO, we found that LTED cells were more sensitive to etomoxir, an inhibitor of CPT-1, the rate-limiting enzyme in β-oxidation of fatty acids. However, these results need to be interpreted with caution, as etomoxir can elicit off-target effects, especially at higher doses such as those used in our studies [62]. In addition, the well-described inverse relationship between fatty acid synthesis and oxidation argues against increased FAO in LTED cells [63, 64].

A recent elegant study by Chen et al. [65] showed that activation of an SREBP-dependent lipogenic program promoted treatment resistance and metastasis in patients with prostate cancer. Of note, SREBPs were reported to regulate a number of other biological pathways such as cell proliferation and differentiation, insulin signaling, and immune response [66–70]. Further studies are required to determine how SREBPs contribute to endocrine resistance in ILC and whether inhibition of SREBP1 would resensitize our LTED models to endocrine therapy.

A potential limitation of our studies is that the generation of LTED cells depends on incubating the cells in CSS, in which the charcoal-stripping removes not only estradiol but also other lipophilic compounds. One could argue that the upregulation of fatty acid and cholesterol metabolism pathways might be a result of decreased lipid levels in CSS. Although this cannot be totally ruled out, we think it is unlikely since *SREBP1* levels were also upregulated in SUM44 TamR cells, which were kept in the same media as their parental cells (serum-free media with supplement of hormones) [13]. Also, our RNA-Seq analysis was performed by using RNA from parental cells kept in CSS for 3 days, thereby removing the potential effect of medium difference between parental and LTED cells on DE gene calling. Thus, we propose that the upregulation of *SREBP1* in LTED cells, and potentially the activation of lipid metabolism, is not caused by the culturing the cells in CSS but instead a mechanism of resistance to estrogen deprivation. Another limitation of our study is that the confirmation in clinical samples was performed using data from a trial that included both patients with IDC and patients with ILC. Currently, there are no gene expression data available from a neoadjuvant trial with sufficiently large numbers of patients with ILC and treatment response data (for example, Ki67) to perform such analyses. An elegant study by Arthur et al. [69] analyzed gene expression in ILC compared with IDC tumors in the neoadjuvant setting; however, the study was limited to responders and thus does not allow comparison of SREBP levels between sensitive and resistant tumors. Finally, our cell line studies were limited to *in vitro* studies at this point in time, and future studies should include mouse models, which would further solidify our findings.

## Conclusions

Our studies provide novel and potentially clinically relevant data on overexpression of and dependency on key enzymes in the fatty acid/cholesterol pathways that collectively suggest a lipogenic reprogramming of metabolism in endocrine-resistant ILC cells. We propose those key enzymes like SREBP1 and FASN as novel targets that deserve future study for the prevention and treatment of endocrine resistance for patients with ILC.

## Additional files

Additional file 1: **Figure S1.** Procedures of generating long-term estrogen deprivation (LTED) cell models. **Figure S2.** Two-dimensional (2D) and three-dimensional (3D) growth of long-term estrogen deprivation (LTED) cells. **Figure S3** Differential expressed (DE) genes in long-term estrogen deprivation (LTED) cells. **Figure S4.** The level of intracellular free cholesterol and cholesterol esters in parental and long-term estrogen deprivation (LTED) cells. **Figure S5.** The expression of sterol regulatory element-binding protein 1c (*SREBP1c*) and *SREBP2* in long-term estrogen deprivation (LTED) cells. **Figure S6.** The maturation processes of sterol regulatory element-binding proteins (SREBPs). **Figure S7.** The expression of enzymes involved in fatty acid synthesis in long-term estrogen deprivation (LTED) cells. **Figure S8.** Dose response of fatty acid synthesis and β-oxidation inhibitors in long-term estrogen deprivation (LTED) cells. **Figure S9.** The abrogation of sterol regulatory element-binding proteins (SREBPs) in SUM44 long-term estrogen deprivation (LTED) cells. **Figure S10.** Expression of sterol regulatory element-binding proteins (*SREBPs*) in clinical samples.

Additional file 2: **Table S1.** Sequences of primers and small interfering RNAs (siRNAs). (XLSX 9 kb)

Additional file 3: **Table S2.** Mapping rate of Salmon. (XLSX 9 kb)

Additional file 4: **Table S3.** Differentially expressed genes in MM134 and SUM44 long-term estrogen deprivation (LTED) cells and SUM44 tamoxifen-resistant cell (SUM44 TamR) cells. (XLSX 3466 kb)

Additional file 5: **Table S4.** Differentially regulated pathways in MM134 and SUM44 long-term estrogen deprivation (LTED) cells. (XLSX 135 kb)

## Abbreviations

2D: Two-dimensional
25-HC: 25-hydroxycholesterol
27-HC: 27-hydroxycholesterol
AI: Aromatase inhibitor
ATCC: American Type Culture Collection
CPT-1: Carnitine palmitoyltransferase 1
CSS: Charcoal-stripped fetal bovine serum
DE: Differential expression
DMEM: Dulbecco’s modified Eagle’s medium
E2: 17β-estradiol
ER: Estrogen receptor
FAO: Fatty acid oxidation
FASN: Fatty acid synthase
FBS: Fetal bovine serum
GEO: Gene Expression Omnibus
GSEA: Gene set enrichment analysis
ICI: ICI 182,780, fulvestrant
IDC: Invasive ductal carcinoma
ILC: Invasive lobular carcinoma
IMEM: Improved minimal essential medium
IPA: Ingenuity Pathway Analysis
LTED: Long-term estrogen deprivation
MM134: MDA-MB-134-VI
mSREBP1: Mature sterol regulatory element-binding protein 1
PBS: Phosphate-buffered saline
PCR: Polymerase chain reaction
Pre-SREBP1: Precursor sterol regulatory element-binding protein 1
RNA-Seq: RNA-sequencing
RXR: Retinoid X receptor
siRNA: Small interfering RNA
SREBP: Sterol regulatory element-binding protein
SUM44 TamR: SUM44 tamoxifen-resistant cell
TPM: Transcript per million
