# Supplementary material for: Key regulators of lipid metabolism drive endocrine resistance in invasive lobular breast cancer
Source: Breast Cancer Res. 2018 Sep 4;20:106. doi: 10.1186/s13058-018-1041-8 (PMC6124012; doi:10.1186/s13058-018-1041-8)
Supplement: Supplementary file 1 — Figure S1. Procedures of generating long-term estrogen deprivation (LTED) cell models. Figure S2. Two-dimensional (2D) and three-dimensional (3D) growth of long-term estrogen deprivation (LTED) cells. Figure S3 Differential expressed (DE) genes in long-term estrogen deprivation (LTED) cells. Figure S4. The level of intracellular free cholesterol and cholesterol esters in parental and long-term estrogen deprivation (LTED) cells. Figure S5. The expression of sterol regulatory element-binding protein 1c (SREBP1c) and SREBP2 in long-term estrogen deprivation (LTED) cells. Figure S6. The maturation processes of sterol regulatory element-binding proteins (SREBPs). Figure S7. The expression of enzymes involved in fatty acid synthesis in long-term estrogen deprivation (LTED) cells. Figure S8. Dose response of fatty acid synthesis and β-oxidation inhibitors in long-term estrogen deprivation (LTED) cells. Figure S9. The abrogation of sterol regulatory element-binding proteins (SREBPs) in SUM44 long-term estrogen deprivation (LTED) cells. Figure S10. Expression of sterol regulatory element-binding proteins (SREBPs) in clinical samples. [file 13058_2018_1041_MOESM1_ESM.pptx]

## Slide 1
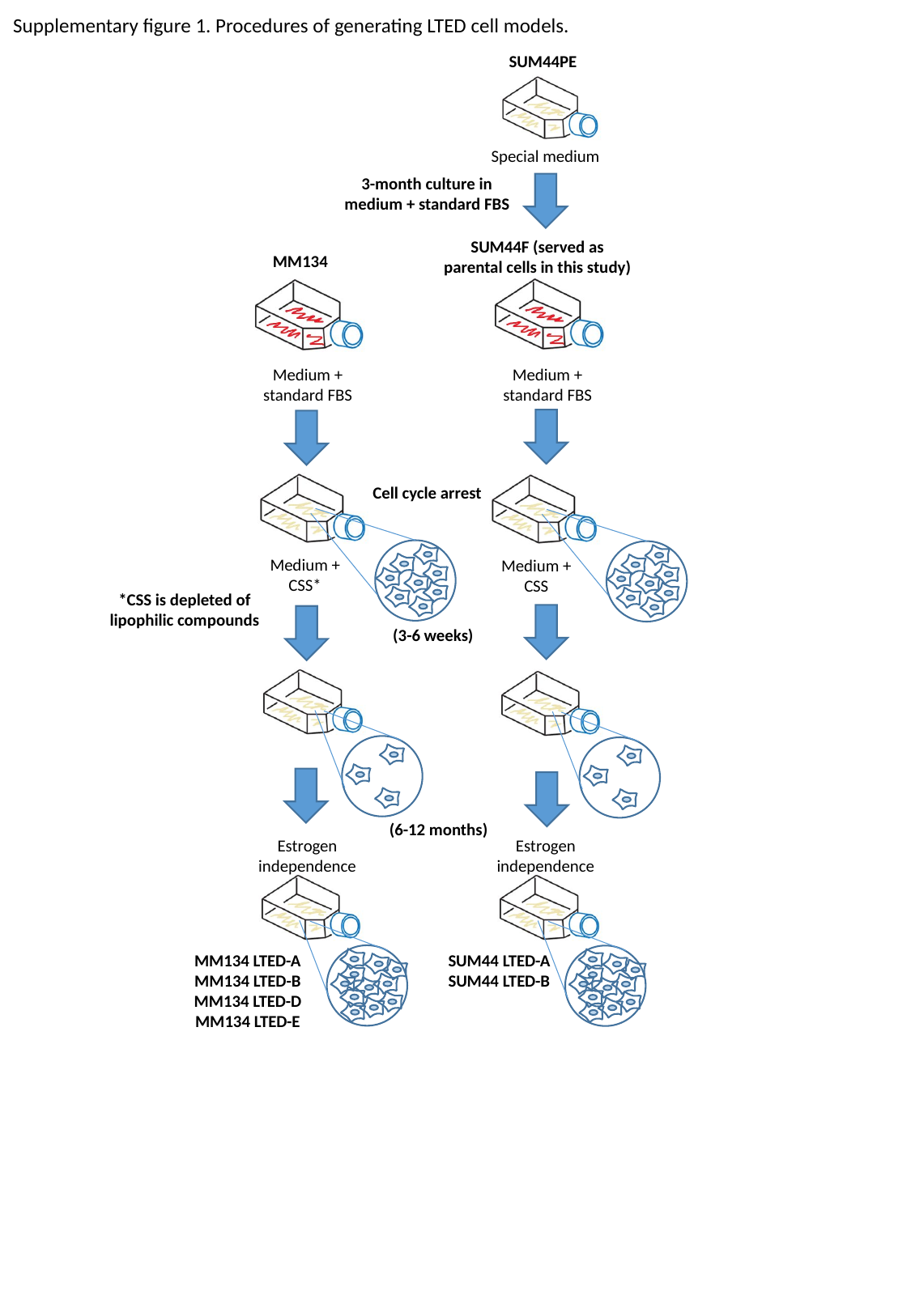

Supplementary figure 1. Procedures of generating LTED cell models.
SUM44PE
Special medium
3-month culture in medium + standard FBS
SUM44F (served as parental cells in this study)
MM134
Medium + standard FBS
Medium + standard FBS
Medium + CSS*
Cell cycle arrest
Medium + CSS
*CSS is depleted of lipophilic compounds
(3-6 weeks)
(6-12 months)
Estrogen
independence
Estrogen
independence
SUM44 LTED-A
SUM44 LTED-B
MM134 LTED-A
MM134 LTED-B
MM134 LTED-D
MM134 LTED-E

## Slide 2
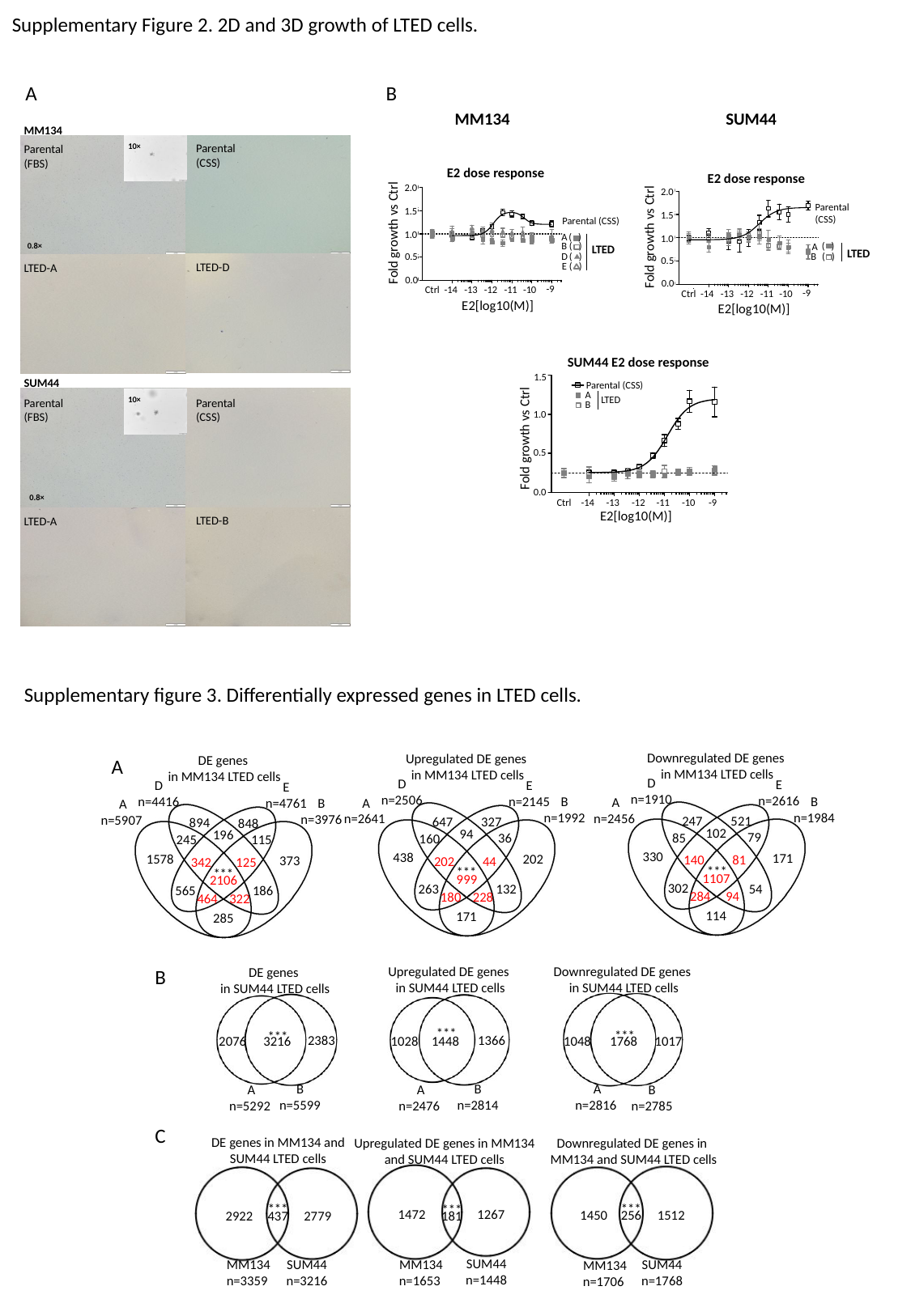

Supplementary Figure 2. 2D and 3D growth of LTED cells.
A
B
MM134
SUM44
MM134
10×
Parental
(CSS)
Parental
(FBS)
E2 dose response
2.0
1.5
Fold growth vs Ctrl
1.0
0.5
0.0
-9
-14
-13
-12
-11
-10
Ctrl
E2[log10(M)]
E2 dose response
2.0
1.5
Fold growth vs Ctrl
1.0
0.5
0.0
-9
-14
-13
-12
-11
-10
Ctrl
E2[log10(M)]
Parental
(CSS)
A
B
LTED
Parental (CSS)
A
B
D
E
( )
( )
LTED
( )
( )
( )
0.8×
( )
LTED-D
LTED-A
SUM44 E2 dose response
1.5
1.0
Fold growth vs Ctrl
0.5
0.0
Ctrl
-13
-12
-11
-10
-9
E2[log10(M)]
-14
SUM44
Parental (CSS)
A
B
LTED
10×
Parental
(FBS)
Parental
(CSS)
0.8×
LTED-B
LTED-A
Supplementary figure 3. Differentially expressed genes in LTED cells.
Downregulated DE genes
in MM134 LTED cells
D
n=1910
E
n=2616
B n=1984
A
n=2456
247
521
102
330
171
81
140
1107
302
54
94
284
114
79
85
***
Upregulated DE genes
in MM134 LTED cells
D
n=2506
E
n=2145
B n=1992
A
n=2641
647
327
94
438
202
44
202
999
263
132
228
180
171
36
160
***
DE genes
in MM134 LTED cells
D
n=4416
E
n=4761
B n=3976
A
n=5907
894
848
196
1578
373
125
342
2106
565
186
322
464
285
115
245
***
A
Downregulated DE genes
in SUM44 LTED cells
1017
1048
1768
A
n=2816
B n=2785
***
Upregulated DE genes
in SUM44 LTED cells
1366
1028
1448
B n=2814
A
n=2476
***
DE genes
in SUM44 LTED cells
2383
2076
3216
B n=5599
A
n=5292
***
DE genes in MM134 and SUM44 LTED cells
2922
437
2779
MM134
n=3359
SUM44 n=3216
***
Upregulated DE genes in MM134 and SUM44 LTED cells
1472
1267
181
SUM44 n=1448
MM134
n=1653
***
Downregulated DE genes in
MM134 and SUM44 LTED cells
1450
1512
256
SUM44 n=1768
MM134
n=1706
***
B
C

## Slide 3
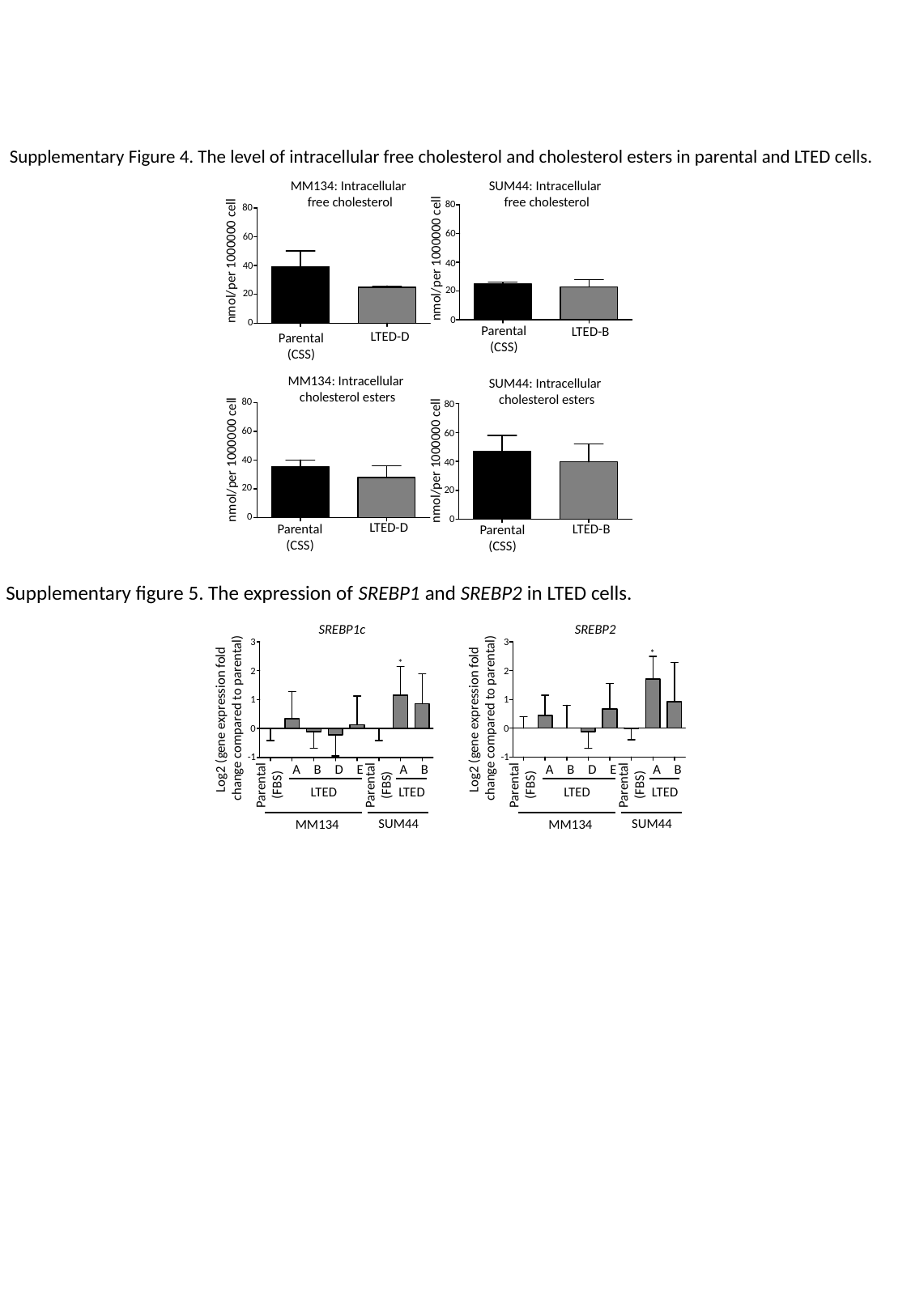

Supplementary Figure 4. The level of intracellular free cholesterol and cholesterol esters in parental and LTED cells.
MM134: Intracellular
free cholesterol
80
60
40
20
0
LTED-D
Parental
(CSS)
nmol/per 1000000 cell
SUM44: Intracellular
free cholesterol
80
60
40
20
0
Parental
(CSS)
LTED-B
nmol/per 1000000 cell
MM134: Intracellular
cholesterol esters
80
60
40
20
0
LTED-D
Parental
(CSS)
nmol/per 1000000 cell
SUM44: Intracellular
cholesterol esters
80
60
40
20
0
LTED-B
Parental
(CSS)
nmol/per 1000000 cell
Supplementary figure 5. The expression of SREBP1 and SREBP2 in LTED cells.
SREBP1c
3
2
1
Log2 (gene expression fold
change compared to parental)
0
-1
A
B
A
B
D
E
Parental
(FBS)
Parental
(FBS)
LTED
LTED
SUM44
MM134
*
SREBP2
3
2
1
Log2 (gene expression fold
change compared to parental)
0
-1
A
B
A
B
D
E
Parental
(FBS)
Parental
(FBS)
LTED
LTED
SUM44
MM134
*

## Slide 4
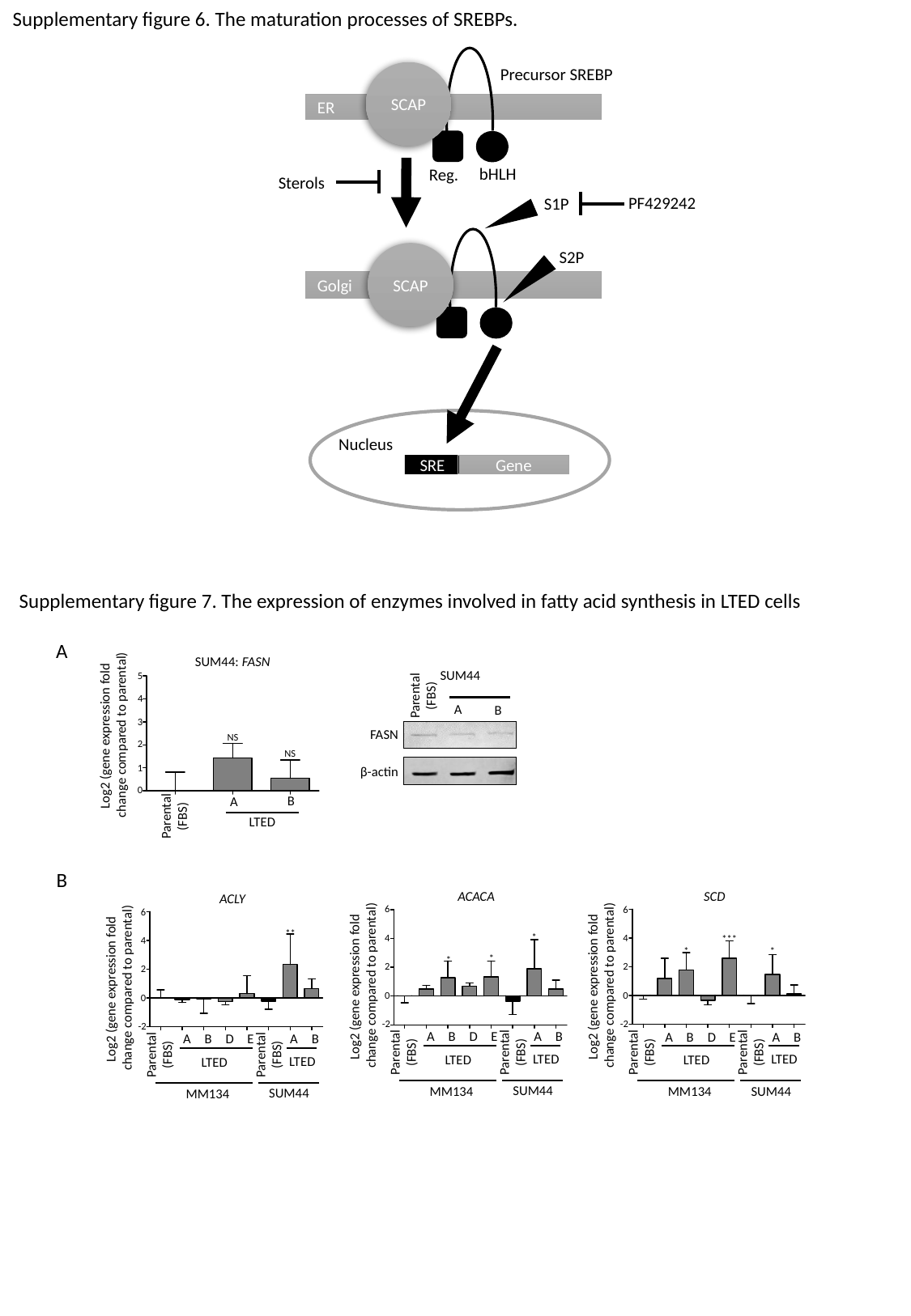

Supplementary figure 6. The maturation processes of SREBPs.
Precursor SREBP
SCAP
ER
bHLH
Reg.
Sterols
S1P
S2P
SCAP
Golgi
Nucleus
SRE
Gene
PF429242
Supplementary figure 7. The expression of enzymes involved in fatty acid synthesis in LTED cells
A
SUM44: FASN
4
3
2
1
0
B
A
Parental
(FBS)
LTED
NS
NS
Log2 (gene expression fold
change compared to parental)
5
SUM44
A
Parental
(FBS)
B
FASN
β-actin
ACACA
6
4
2
Log2 (gene expression fold
change compared to parental)
0
-2
A
B
A
B
D
E
Parental
(FBS)
Parental
(FBS)
LTED
LTED
SUM44
MM134
*
*
SCD
6
4
2
Log2 (gene expression fold
change compared to parental)
0
-2
A
B
A
B
D
E
Parental
(FBS)
Parental
(FBS)
LTED
LTED
SUM44
MM134
*
***
*
ACLY
6
4
2
Log2 (gene expression fold
change compared to parental)
0
-2
A
B
A
B
D
E
Parental
(FBS)
Parental
(FBS)
LTED
LTED
SUM44
MM134
**
*
B

## Slide 5
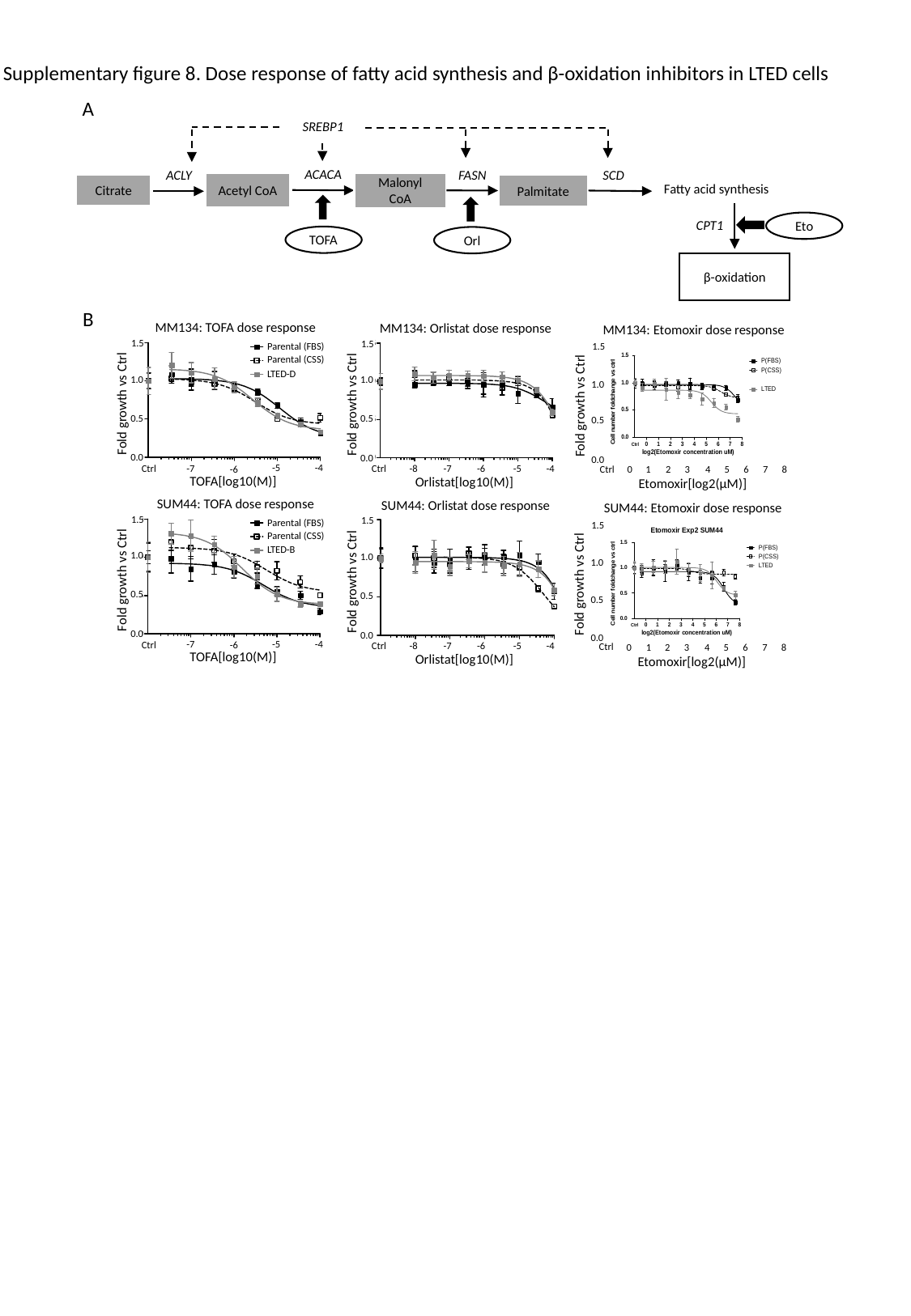

Supplementary figure 8. Dose response of fatty acid synthesis and β-oxidation inhibitors in LTED cells
A
SREBP1
ACACA
ACLY
FASN
Malonyl CoA
Acetyl CoA
Palmitate
Citrate
Fatty acid synthesis
TOFA
β-oxidation
SCD
CPT1
Eto
Orl
B
MM134: TOFA dose response
1.5
1.0
Fold growth vs Ctrl
0.5
0.0
-4
-5
-7
Ctrl
-6
TOFA[log10(M)]
Parental (FBS)
Parental (CSS)
LTED-D
MM134: Orlistat dose response
1.5
1.0
Fold growth vs Ctrl
0.5
0.0
-7
-5
-4
-8
Ctrl
Orlistat[log10(M)]
-6
MM134: Etomoxir dose response
1.5
1.0
Fold growth vs Ctrl
0.5
0.0
Ctrl
0
Etomoxir[log2(µM)]
4
5
6
7
8
1
2
3
SUM44: TOFA dose response
1.5
1.0
Fold growth vs Ctrl
0.5
0.0
-4
-5
-7
Ctrl
-6
TOFA[log10(M)]
Parental (FBS)
Parental (CSS)
LTED-B
SUM44: Orlistat dose response
1.5
1.0
Fold growth vs Ctrl
0.5
0.0
-7
-5
-4
-8
Ctrl
Orlistat[log10(M)]
-6
SUM44: Etomoxir dose response
1.5
1.0
Fold growth vs Ctrl
0.5
0.0
Ctrl
0
Etomoxir[log2(µM)]
4
5
6
7
8
1
2
3

## Slide 6
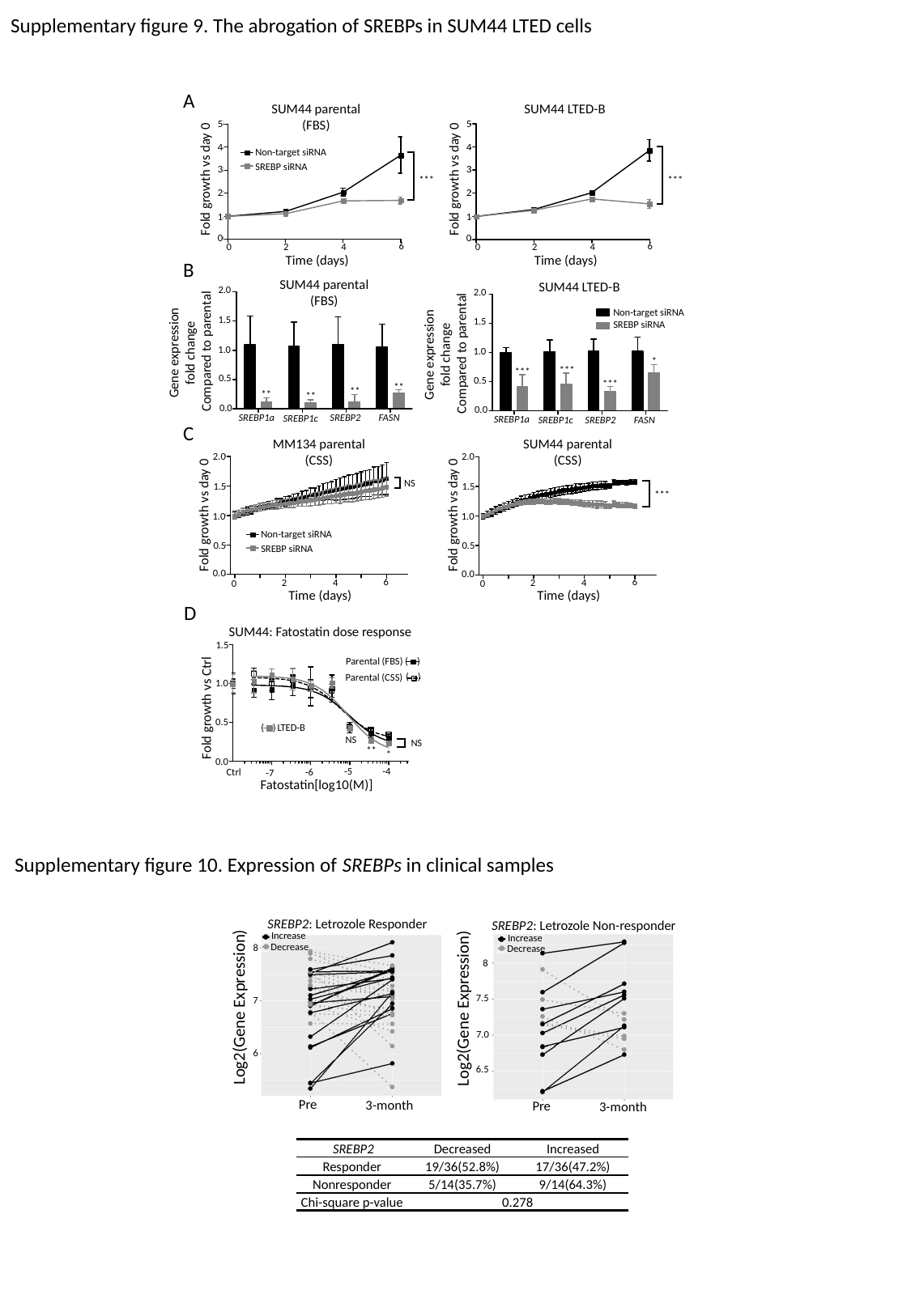

Supplementary figure 9. The abrogation of SREBPs in SUM44 LTED cells
A
SUM44 parental (FBS)
5
3
Fold growth vs day 0
2
1
0
6
0
4
2
Time (days)
***
4
SUM44 LTED-B
5
3
Fold growth vs day 0
2
1
0
6
0
4
2
Time (days)
***
4
Non-target siRNA
SREBP siRNA
B
SUM44 parental (FBS)
2.0
1.5
Gene expression
fold change
Compared to parental
1.0
0.5
**
**
**
**
0.0
SREBP1a
SREBP2
FASN
SREBP1c
SUM44 LTED-B
2.0
1.5
Gene expression
fold change
Compared to parental
1.0
*
***
***
0.5
***
0.0
SREBP1a
SREBP2
FASN
SREBP1c
Non-target siRNA
SREBP siRNA
C
MM134 parental (CSS)
2.0
1.5
Fold growth vs day 0
1.0
0.5
0.0
6
2
4
0
Time (days)
NS
SUM44 parental (CSS)
2.0
1.5
Fold growth vs day 0
1.0
0.5
0.0
6
2
4
0
Time (days)
***
Non-target siRNA
SREBP siRNA
D
SUM44: Fatostatin dose response
1.5
1.0
Fold growth vs Ctrl
0.5
0.0
-4
-5
-6
Ctrl
-7
Fatostatin[log10(M)]
( )
Parental (FBS)
( )
Parental (CSS)
( )
LTED-B
NS
NS
**
*
Supplementary figure 10. Expression of SREBPs in clinical samples
SREBP2: Letrozole Responder
Increase
Decrease
7
6
Pre
3-month
8
Log2(Gene Expression)
SREBP2: Letrozole Non-responder
Increase
Decrease
7.5
7.0
6.5
Pre
3-month
8
Log2(Gene Expression)
| SREBP2 | Decreased | Increased |
| --- | --- | --- |
| Responder | 19/36(52.8%) | 17/36(47.2%) |
| Nonresponder | 5/14(35.7%) | 9/14(64.3%) |
| Chi-square p-value | 0.278 | |

## Slide 7
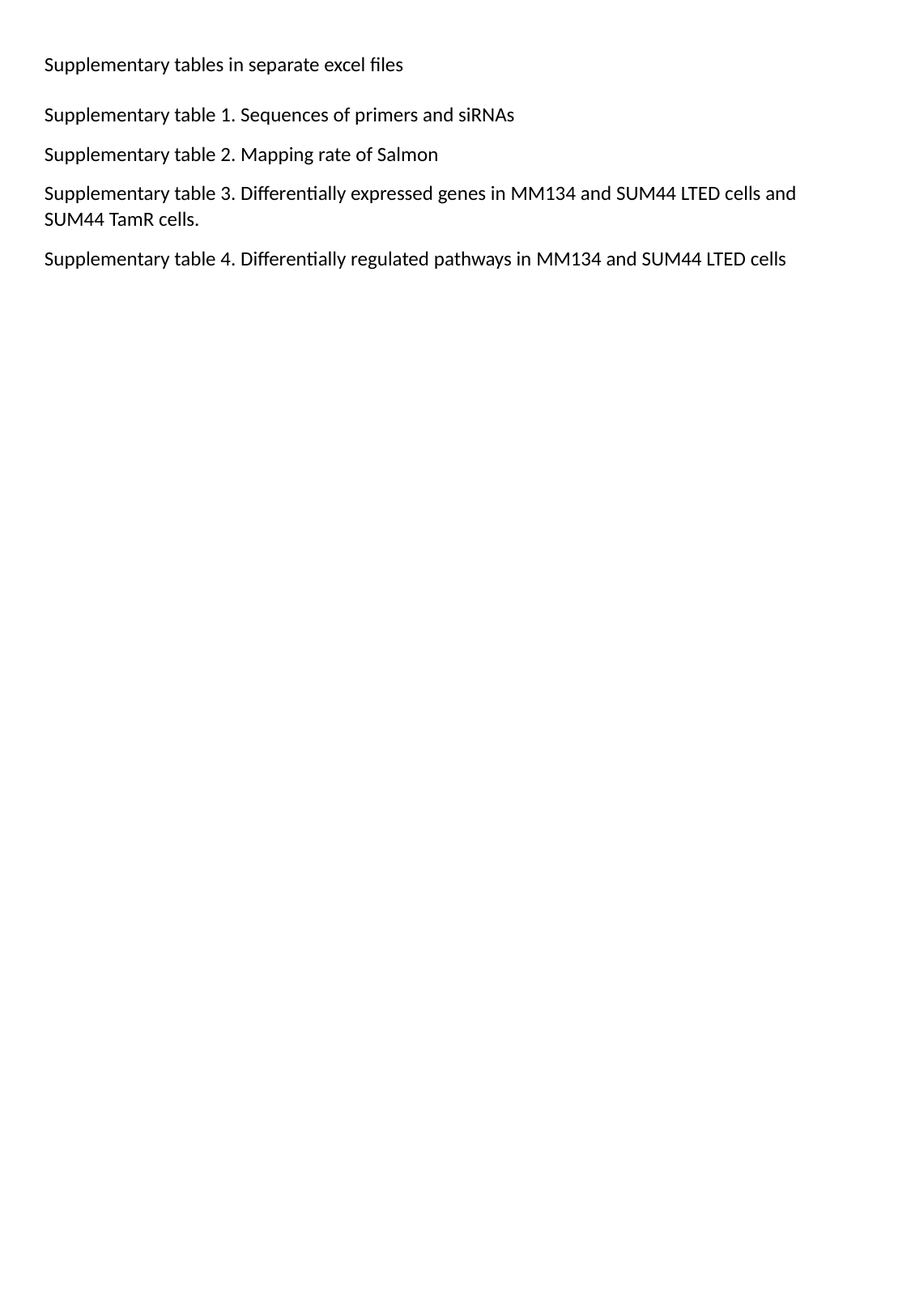

Supplementary tables in separate excel files
Supplementary table 1. Sequences of primers and siRNAs
Supplementary table 2. Mapping rate of Salmon
Supplementary table 3. Differentially expressed genes in MM134 and SUM44 LTED cells and SUM44 TamR cells.
Supplementary table 4. Differentially regulated pathways in MM134 and SUM44 LTED cells
